# Global longitudinal strain manually measured from mid-myocardial lengths is a reliable alternative to speckle tracking global longitudinal strain

**DOI:** 10.1186/s44348-024-00038-x

**Published:** 2024-11-19

**Authors:** Chee Cheen Yeong, Danielle L. Harrop, Arnold C. T. Ng, William Y. S. Wang

**Affiliations:** 1 Department of Cardiology, Princess Alexandra Hospital, Brisbane, QLD Australia; 2 Faculty of Medicine, The University of Queensland, Brisbane, QLD Australia

**Keywords:** Global longitudinal strain, Echocardiography, Left ventricular function,, Cardiac Imaging Techniques, Heart Failure

## Abstract

**Background:**

Global longitudinal strain (GLS) is a useful marker for the echocardiographic evaluation of left ventricular (LV) systolic dysfunction. Presently GLS is derived from speckle tracking of LV images, but speckle tracking software is not always available. We seek to determine if manually measured GLS (MM-GLS) by assessing mid-myocardial lengths can be a reliable alternative to speckle tracking GLS (ST-GLS).

**Methods:**

Transthoracic echocardiogram images of a tertiary hospital in Australia were retrospectively analyzed to study the relationships between ST-GLS, MM-GLS, and LV ejection fraction (LVEF). We further evaluated the impact of image quality and regional wall motion abnormalities on those relationships.

**Results:**

Echocardiography studies from 154 patients were included (female sex, 36%; mean age, 61.7 ± 14.8 years). The average LVEF was 51.3% ± 11.3% and the average ST-GLS was 16.7 ± 3.8. MM-GLS strongly correlated with ST-GLS (intraclass correlation coefficient, 0.986; *P* < 0.001) and with LVEF regardless of the presence of regional wall motion abnormalities. If using GLS cutoff of more than 18% as normal, 97.5% of studies with normal ST-GLS had normal MM-GLS. If using GLS cutoff as less than 16% as abnormal, 95.5% of studies with abnormal ST-GLS had abnormal MM-GLS. There was no case with ST-GLS > 18% and MM-GLS < 16%, nor were there any case in with ST-GLS < 16% and MM-GLS > 18%.

**Conclusions:**

MM-GLS correlates strongly with ST-GLS. If ST-GLS cannot be accurately assessed, MM-GLS may be a useful alternative to provide GLS values in both clinical and research studies.

**Supplementary Information:**

The online version contains supplementary material available at 10.1186/s44348-024-00038-x.

## Background

Global longitudinal strain (GLS), based on speckle tracking echocardiography, assesses the longitudinal deformation of the left ventricle (LV). It has been shown to be a useful and reliable measurement in evaluating early LV systolic dysfunction [1, 2].

GLS is the average proportional longitudinal LV myocardial deformation in end-systole compared to end-diastole [3, 4]. At present, the measurement of LV longitudinal strain values in echocardiography uses computerized speckle tracking of echo signals in progressive images. The strain values of each myocardial segment are calculated, and averages the values from the apical four-, two-, and three-chamber views are derived and then averaged to provide speckle tracking GLS (ST-GLS). Measuring ST-GLS requires the use of specialized speckle tracking software which is presently not universally available. The measurement of GLS based on speckle tracking is not standardized between different ultrasound machine vendors [5]. At present, there is no manual method to check the accuracy of GLS provide by speckle tracking echocardiography on an individual basis.

Strain is the ratio of the change of length compared to its original length and could therefore be measured whenever lengths could be measured. Assessing changes in mid-myocardial lengths, Kobayashi et al. [6] showed that longitudinal strain measurements manually derived from apical four-chamber views correlated well with values derived using speckle tracking. Okada et al. [7] noted that longitudinal strain values manually measurements on apical four- and two-chamber views also correlated with speckle tracked values. However, the calculation of GLS requires the combined analysis of values derived from apical four-, two- and three-chamber images. We evaluate the association between manually measured GLS (MM-GLS) and ST-GLS by using images from these views.

## Methods

### Study population

The study cohort consisted of 154 consecutive patients who had echocardiogram performed in the month of July 2020 at a single Australian tertiary hospital using a GE Vivid E9 Ultrasound System (GE Vingmed). Both inpatients and outpatients with complete transthoracic echocardiogram were included in this study.

### Echocardiography

All study subjects underwent a transthoracic echocardiogram examination at rest in the left lateral decubitus position with a Vivid E9 ultrasound system. Images and measurements were acquired according to the recommendations of the American Society of Echocardiography. LV end-diastolic volume and LV end-systolic volume were indexed to body surface area. LVEF was assessed via Simpson biplane method and reported by a consultant cardiologist.

### Image quality

Image quality, defined as the extent of visualization of the endocardium, was graded 0 up to 4 based on the following: grade 0, no useful or interpretable images; grade 1, poor images, gross structures visible but no details; grade 2, diagnostic images but missing details; grade 3, good images; and grade 4, perfect quality images with all fine details clearly visible.

### Two-dimensional speckle tracking echocardiography

**Fig. 1:**
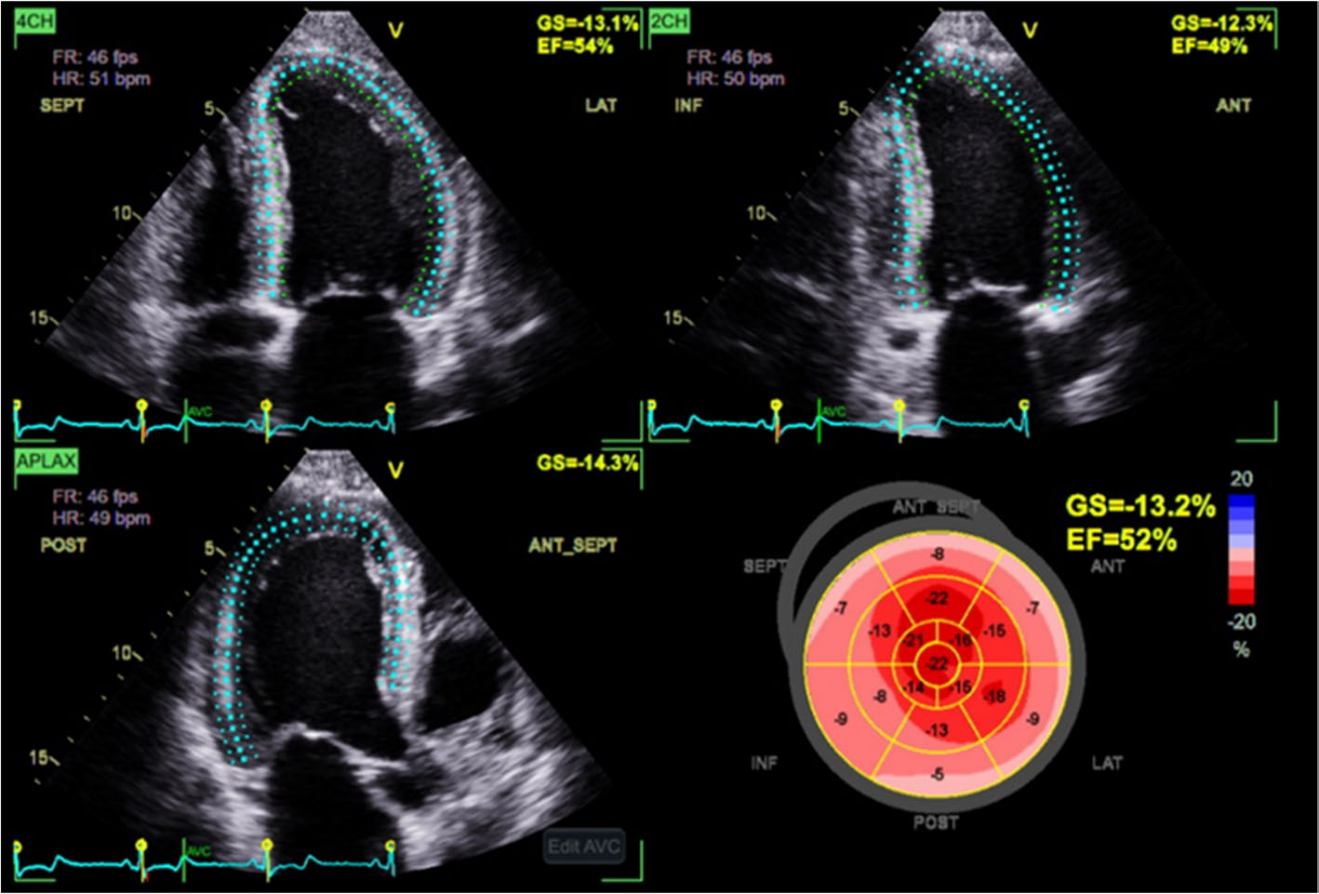
Example of GLS measured by speckle tracking

ST-GLS was measured offline using dedicated software (EchoPac ver. 113, GE Vingemed). For each four, two, and three chamber views, the LV cavity was divided into six segments (basal, mid, and apical parts of two opposing walls) and strain curves were generated for each segment. ST-GLS was then calculated as the average of values evaluated from each view and provided by the inbuilt software (Fig. 1). GLS is frequently expressed as a negative value with the negativity to imply length reduction, but other studies have expressed it as a positive absolute value. In this study, we will present GLS as positive values with shortening being positive.

### Manual measurement of GLS

MM-GLS was evaluated for all 154 study participants. For four- and two-chamber views, the mid-myocardial length of the LV cavity was measured from one end of the base of the mitral annulus to the apex and then to the other mitral annulus. For the three-chamber view, the mid-myocardial length of the LV cavity was measured from the base of the mitral annulus, to the apex, and then to the basal end of the interventricular septum. The most representative beat in each view was selected for each patient. The diastolic mid-myocardial length (MMLdia) was measured at end-diastole. The systolic mid-myocardial length (MMLsys) was measured at peak systole.

The change in fractional LV mid-myocardial length (%) was calculated as “(systolic mid-myocardial length – diastolic mid-myocardial length) / diastolic mid-myocardial length × 100.”

**Fig. 2:**
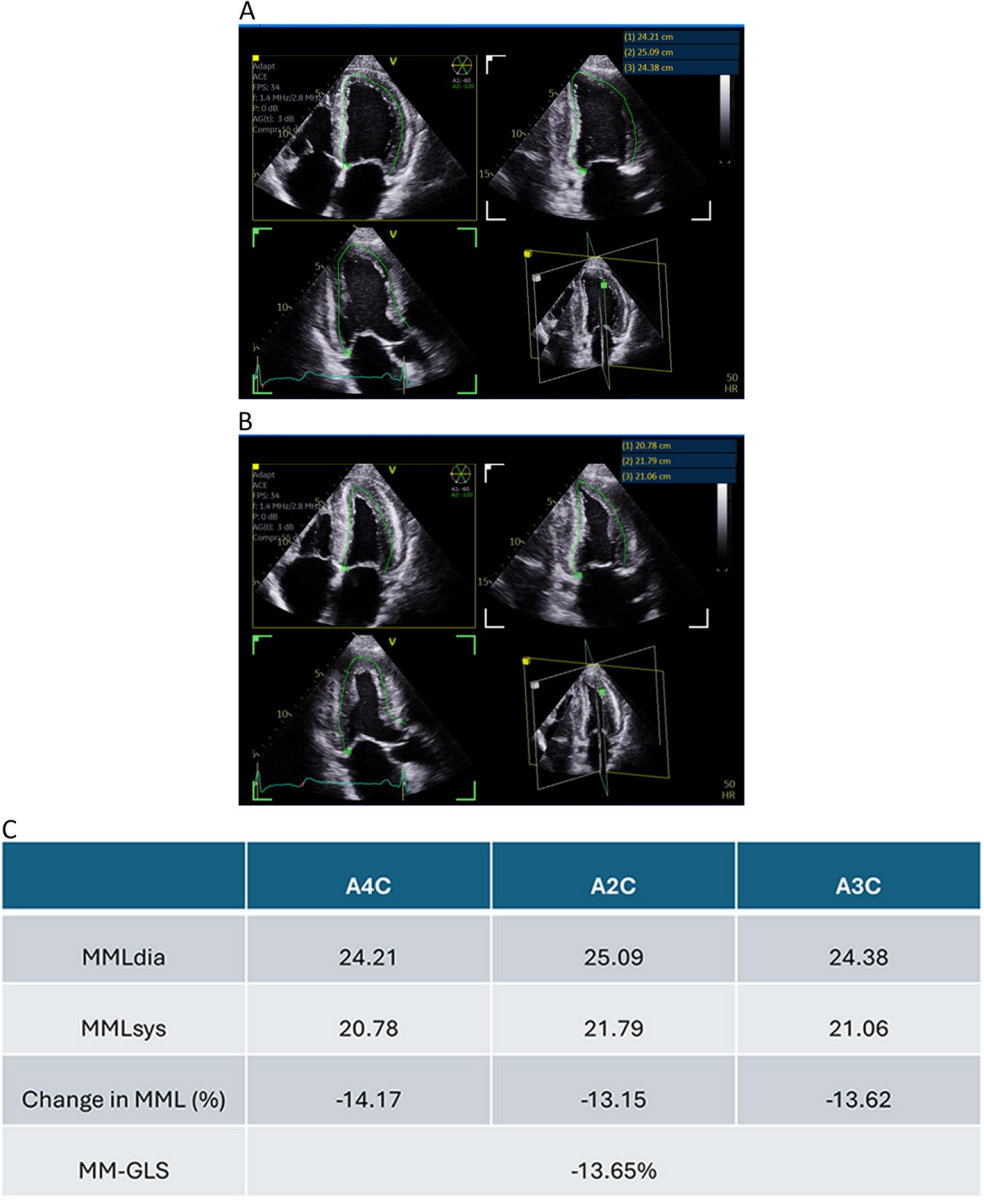
Example of GLS measured manually (MM-GLS). Measurements of (**a**) diastolic mid-myocardial length, measurement of (**b**) systolic mid-myocardial length, and (**c**) calculation of MM-GLS

Measurements in the three apical views were then averaged to obtain the final product of manually measured GLS (Fig. 2).

ST-GLS and MM-GLS were measured more than 3 months apart for the whole cohort by same investigator, blinded to the result of the previous measurements. A subset of 25 cases were selected to measure intraobserver and interobserver reproducibilities for MM-GLS.

### Statistical analysis

Continuous variables were tested for normal distribution using the Kolmogorov–Smirnov test and presented as mean ± standard deviation if normally distributed. Categorical data was presented as frequencies and percentages. Pearson correlation was used to examine the linear association between two continuous variables. Comparisons of two proportions were performed using chi-square test or Fisher exact test as appropriate. Intraclass correlation coefficients (ICCs) were calculated for interobserver and intraobserver strains, and consistency between the strain methods. All analyses were performed with IBM SPSS ver. 24.0 (IBM Corp), and a two-tailed *P* < 0.05 was considered statistically significant.

## Results

### Patient demographics and echocardiographic parameters

All 154 study participants were eligible for GLS analysis. Table 1 shows patient demographics and echocardiographic parameters. Table 2 shows the intraobserver and interobserver reproducibilities for ST-GLS and MM-GLS. Bland–Altman plots for MM-GLS intraobserver and interobserver reproducibilities are shown in Figs. S1 and S2, respectively. The mean time to perform ST-GLS was 3 min and 5 s, while the median was 2 min and 59 s (interquartile range, 2 min and 21 s to 3 min and 41 s). The mean time to perform MM-GLS was 4 min and 43 s, and the median time was 4 min and 47 s (interquartile range, 4 min to 5 min and 25 s).

**Table 1:** Baseline demographics and echocardiographic parameters

Characteristic	Value (*n* = 154)
Age (yr)	61.7 ± 14.75
Sex	
Male	98 (63.6)
Female	56 (36.4)
Body surface area (m^2^)	1.96 ± 0.23
Systolic blood pressure (mmHg)	129 ± 17
Diastolic blood pressure (mmHg)	65 ± 14
Heart rate (bpm)	66 ± 15
Hypertension	112 (72.7)
Diabetes	104 (67.5)
Dyslipidemia	97 (63.0)
Smoker/ex-smoker	106 (68.8)
Echocardiographic parameter	
LV mass index (g/m^2^)	89.63 ± 30.42
LV end-diastolic diameter indexed (mL/m^2^)	67.8 ± 23.2
LV end-systolic diameter indexed (mL/m^2^)	35.33 ± 18.52
LVEF (%)	51.32 ± 11.3
Left atrial volume index (mL/m^2^)	39.8 ± 16.9
E/A	1.08 ± 0.58
Deceleration time (msec)	211.31 ± 58.4
E/e’ (cm/sec)	10.69 ± 5.54
Speckle tracking GLS	16.72 ± 3.75
Presence of RWMAs	35 (22.7)

Values are presented as mean ± standard deviation or number (%)

bpm, beats per minute; *LV* left ventricle, *LVEF* left ventricular ejection fraction, *E/A* ratio of E to peak atrial systolic transmitral flow velocity, *E/e’* ratio of peak early-diastolic transmitral flow velocity to peak early-diastolic mitral annular velocity at septal annulus, *GLS* global longitudinal strain; *RWMA* regional wall motion abnormality

**Table 2:** Intraobserver and interobserver reproducibilities

Variable	Intraobserver			Interobserver		
	ICC	95% CI	*P*-value	ICC	95% CI	*P*-value
ST-GLS	0.980	0.955–0.991	< 0.001	0.967	0.916–0.986	< 0.001
MM-GLS	0.975	0.943–0.989	< 0.001	0.966	0.924–0.985	< 0.001

*ICC* intraclass correlation coefficient, *CI* confidence interval *ST-GLS* speckle tracking global longitudinal strain, *MM-GLS* manually measured global longitudinal strain

Primary indications for echocardiography included assessments for chest pains or coronary artery disease (*n* = 35, 22.7%), heart valves and aorta (*n* = 32, 20.8%), chemotherapy (*n* = 28, 18.2%), potential endocarditis (*n* = 14, 9.1%), heart failure (*n* = 13, 8.4%), stroke (*n* = 10, 6.5%), pulmonary hypertension (*n* = 10, 6.5%), arrhythmias (*n* = 7, 4.5%), infiltrative disease (*n* = 4, 2.6%), and pericardial disease (*n* = 1, 0.6%). Forty-five patients (29.2%) had LVEF of < 50%.

### Image quality

Based on the abovementioned grading system for image quality, there were 49 studies (32%) classified as having lower image quality including 2 (1.3%) in grade 0, 2 (1.3%) in grade 1, and 45 (29.2%) in grade 2. There were 105 studies (68.2%) classified as having higher image quality, with 97 (63.0%) in grade 3, and 8 (5.2%) in grade 4.

### ST-GLS vs. MM-GLS

**Fig. 3:**
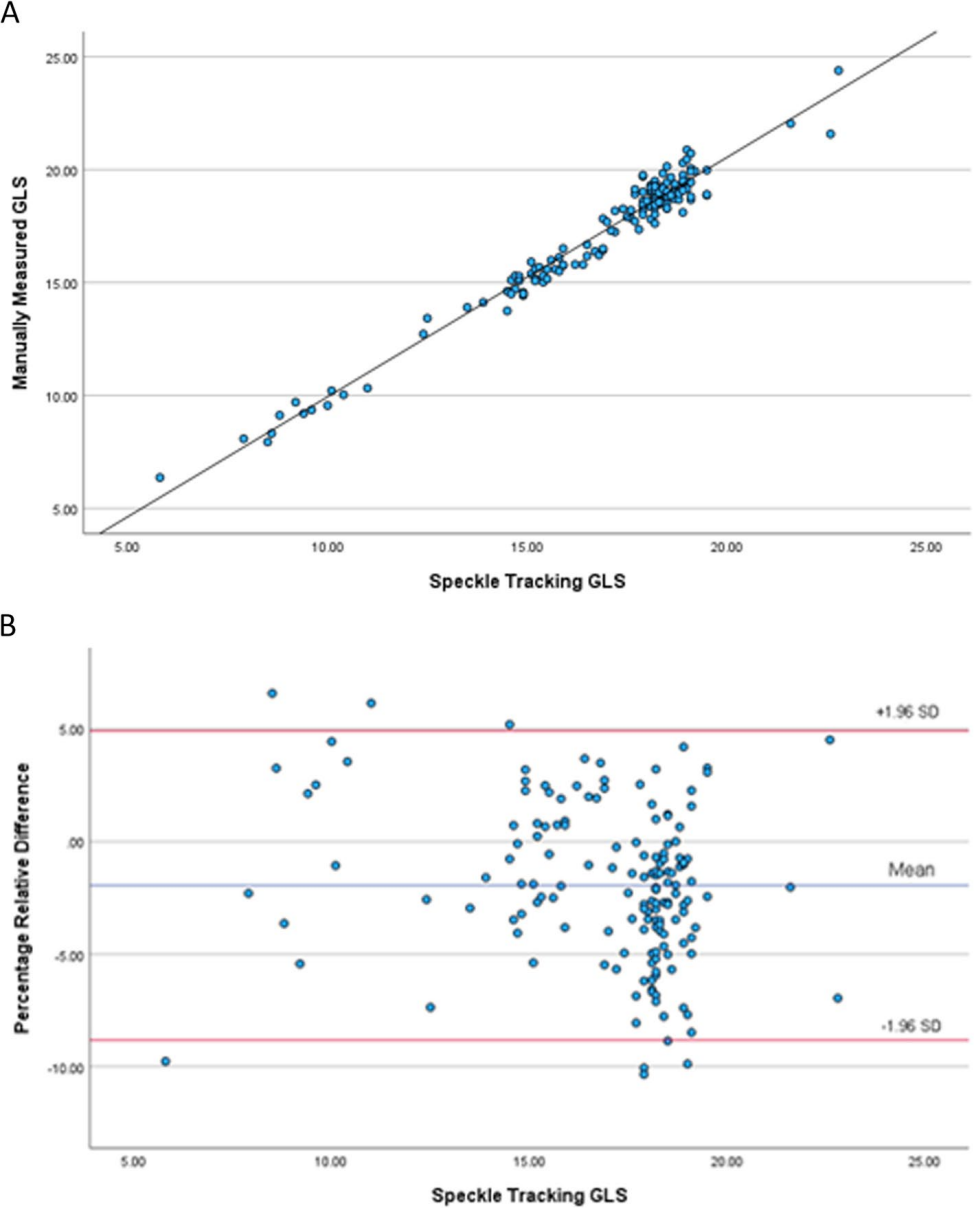
Correlation analyses of speckle tracking GLS and manually measured GLS (**A**) and Bland–Altman Plot (**B**)

There was a high degree of correlation between ST-GLS and MM-GLS (ICC, 0.986; *P* < 0.001) (Fig. 3). The average proportional difference between ST-GLS and MM-GLS, defined as “(ST-GLS – MM-GLS) / ST-GLS,” was 0.0196. ST-GLS and MM-GLS correlated with each other in both the higher image quality subgroup (*n* = 105; ICC, 0.986; *P* < 0.001) and the lower quality subgroup (*n* = 49; ICC, 0.986; *P* < 0.001). ST-GLS and MM-GLS correlated with each other in subjects with normal/preserved LVEF of ≥ 50% (*n* = 109; ICC, 0.897; *P* < 0.001) and in subjects with impaired LVEF of < 50% (*n* = 45; ICC, 0.995; *P* < 0.001).

**Fig. 4:**
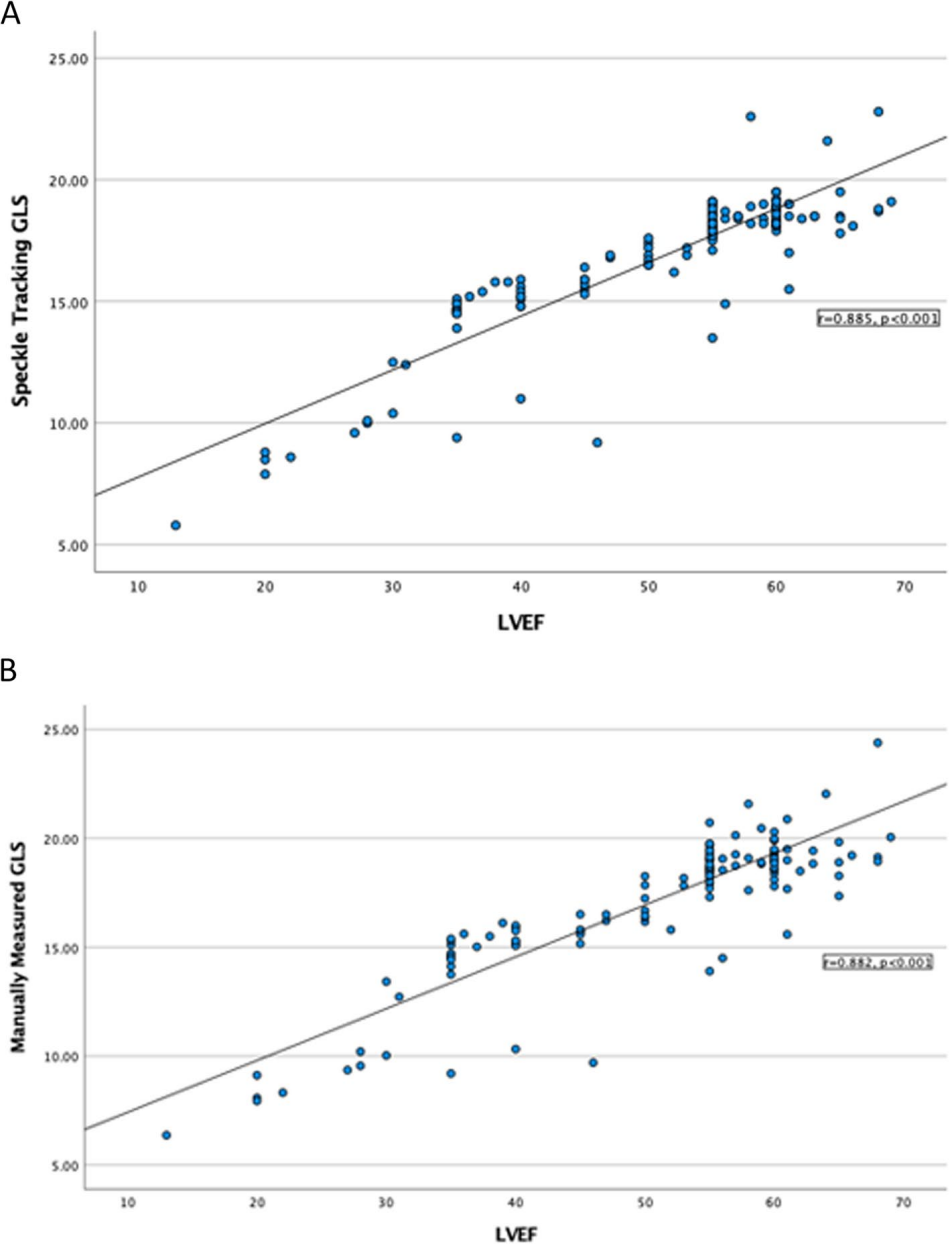
Correlation analyses of LVEF and (**a**) Speckle Tracking GLS, (**b**) manually calculated GLS

ST-GLS correlated strongly with LVEF (r = 0.885, *P* < 0.001) (Fig. 4A). MM-GLS also correlated strongly with LVEF (r = 0.838, *P* < 0.001) (Fig. 4B). The strong correlations between LVEF, ST-GLS, and MM-GLS were not affected by image quality with significant correlations for all comparisons in both lower and higher image quality subgroups (*P* < 0.001). There were 35 subjects (22.7%) with LV regional wall motion abnormalities (RWMAs). ST-GLS and MM-GLS correlated with each other in studies with RWMAs (ICC, 0.992; *P* < 0.001) and without RWMAs (ICC, 0.971; *P* < 0.001).

If using GLS cutoff of greater than 18% as normal [8], 97.5% and 100% of subjects with normal ST-GLS had MM-GLS of > 18% and ≥ 16%, respectively (Table 3). If using GLS cutoff of less than 16% as abnormal [8], 95.5% and 100% of subjects with abnormal ST-GLS < 16% had MM-GLS of < 16% and ≤ 18%, respectively (Table 4). We did not observe any case with ST-GLS > 18% and MM-GLS < 16%, nor did we observe any case in with ST-GLS < 16% and MM-GLS > 18%.

**Table 3:** Distribution of ST-GLS and MM-GLS with cutoff of > 18% as normal (*n* = 154)

		MM-GLS	
		≤ 18%	> 18%
ST-GLS	≤ 18% (*n* = 75)	60 (80.0)	15 (20.0)
	> 18% (*n* = 79)	2 (2.5)	77 (97.5)

Chi-square test, χ^2^(1) = 96; *P* < 0.001

*ST-GLS* speckle tracking global longitudinal strain, *MM-GLS* manually measured global longitudinal strain

**Table 4:** Distribution of ST-GLS and MM-GLS with cutoff of < 16% as abnormal (*n* = 154)

		MM-GLS	
		< 16%	≥ 16%
ST-GLS	< 16% (*n* = 44)	42 (95.5)	2 (4.5)
	≥ 16% (*n* = 110)	2 (1.8)	108 (98.2)

Chi-square test, χ^2^(1) = 135; *P* < 0.001

*ST-GLS* speckle tracking global longitudinal strain, *MM-GLS*, manually measured global longitudinal strain

## Discussion

In this study, we have shown that MM-GLS derived using four-, two- and three-chamber apical views correlated strongly with ST-GLS and LVEF. These associations were not affected by image quality or the presence of RWMAs. To our knowledge, this is the first study to directly compare ST-GLS and MM-GLS based on apical four-, two-, and three-chamber images. Previous studies on manually measured strain did not utilized all three apical images and therefore not provided GLS [6, 7].

The level of absolute correlation we observed between MM-GLS and ST-GLS was extremely strong (ICC, 0.986; *P* < 0.001), indicating that MM-GLS can serve as a surrogate for ST-GLS. MM-GLS can be used to check the result of ST-GLS if individual operators have doubts about the ST-GLS result. This could be especially helpful in studies which part or whole of the LV were difficult to assess providing suboptimal images, where it would be difficult to ascertain the reliability of data computed into the software for GLS.

Compared to LVEF, ST-GLS has an array of applications and offers additional information. ST-GLS can help differentiate etiology of left ventricular hypertrophy, detect early subclinical myocardial dysfunction before LVEF and used for monitoring patients receiving chemotherapy [9]. ST-GLS can be a sensitive early marker of LV dysfunction, with a decline in GLS preceding a decrease in LVEF [9–11].

Recently, Haji et al. [2] showed that the incorporation of GLS as part of the criteria for early-stage heart failure may enables the recognition of more patients at risk and help predict heart failure admissions. In that study, GLS of > 18% was defined as normal, GLS of < 16% was defined as abnormal, and 16% to 18% was regarded as borderline. Our results here show that those cutoffs, the great majority of study subjects with GLS > 18% or < 16% measured on ST-GLS will be allocated to the same categories if measured using MM-GLS (Tables 3, 4). Furthermore, we did not observe any case in which ST-GLS was normal and MM-GLS was abnormal, neither did we observe any case in which ST-GLS was abnormal and MM-GLS was normal. These results confirm the potential use of MM-GLS as a substitute for ST-GLS if necessary.

While speckle tracking has the advantages of measuring strain values progressively from one image to the next across the entire cardiac cycle, most of that information is not utilized in the calculation of GLS since GLS is based on peak end-diastole and end-systole values. Furthermore, while speckle tracking allows the measurement of strain in different cardiac segments, which is useful in disease such as cardiac amyloidosis [12, 13], the segmental information is lost when GLS is calculated as GLS is based on averages. Therefore, it is not surprising that ST-GLS correlates highly with MM-GLS, despite ST-GLS uses significantly more data input. While the measuring MM-GLS using mid-LV myocardial length was done manually in this study, it should be possible to program the method to be done automatically.

Due to intervendor software variability of ST-GLS, serial assessments of ST-GLS in patients should ideally be performed using the same equipment and software. Different vendors have different cutoffs for a “normal” ST-GLS. For example, GE platform has a reference range of 18.36 ± 1.45, Philips platform has a reference of range of 17.09 ± 1.96, and Toshiba platform has a reference range of 16.39 ± 1.52 [5]. These differences may provide issues in clinical practice unless sequential studies are performed on the same platform.

Although the use of GLS in clinical practice is becoming more prevalent, ST-GLS requires the additional advanced speckle tracking software potentially at additional financial cost. This raises concerns in health equity due to potential impact on healthcare delivery in financially less resourced countries and services. On the other hand, the measurement of MM-GLS does not require any advanced software package and can potentially be assessed by any software that allows the measurement of length.

Image quality is an integral variable that could affect observer and measurement variability. It has been reported that 10% to 15% of routine echocardiograms have poor image quality [14]. Experience of the sonographer and software advancement of the ultrasound imaging system are two main factors that affect quality of the acquired images [15]. Poor image quality affects both the assessment of LVEF and ST-GLS. We have demonstrated that the correlation between MM-GLS, ST-GLS, and LVEF are high in both the higher and lower image quality groups. However, the majority of our study subjects in the lower image quality group had grade 2 image quality and there were only four study subjects with grades 0 or 1 image quality. Therefore, we cannot be certain about the usefulness of MM-GLS in in subjects with very poor (grade 0 or 1) image quality.

Measurement of strain in the other cardiac chambers have received increasing interest [16, 17]. However, speckled tracking of the thin-walled cardiac chambers such as atria and right ventricle are more challenging compared to speckle tracking for the LV [18]. The measurement of length shortening using MM-GLS like methods could potentially be useful for cardiac chambers.

This study has several limitations. First, this is a single-center study with moderate sample size. Second, LVEF values were calculated based on echocardiogram images using Simpson biplane method. This method is known to have issues with variability. Measuring LVEF using an alternative modality such as cardiac magnetic resonance imaging may provide more consistent and reliable results. Third, MM-GLS does not measure focal strain patterns. The knowledge of focal strain patterns can be important in diseases such as amyloidosis, Fabry disease, and ischemic heart disease. Last but not least, changes in ST-GLS have shown to be associated with clinical outcomes data. Although we have demonstrated in this study that MM-GLS correlates strongly with ST-GLS, we have not yet demonstrated relationships between MM-GLS and clinical outcomes.

## Conclusions

MM-GLS correlates strongly with ST-GLS and could potentially be used as a reliable alternative if ST-GLS is unavailable or cannot be accurately assessed.

## Supplementary Information

Additional file 1: Fig. S1. Bland-Altman plot for intraobserver reproducibilities of MM-GLS. Fig. S2. Bland-Altman plot for interobserver reproducibilities of MM-GLS.

## Data Availability

No datasets were generated or analysed during the current study.

## Abbreviations

GLS: Global longitudinal strain
ICC: Intraclass correlation coefficient
LV: Left ventricle
LVEF: Left ventricular ejection fraction
MM-GLS: Manually measured global longitudinal strain
RWMA: Regional wall motion abnormality
ST-GLS: Speckle tracking global longitudinal strain
