# Supplementary material for: Global longitudinal strain manually measured from mid-myocardial lengths is a reliable alternative to speckle tracking global longitudinal strain
Source: J Cardiovasc Imaging. 2024 Nov 19;32:35. doi: 10.1186/s44348-024-00038-x (PMC11575028; doi:10.1186/s44348-024-00038-x)

Supplementary Figure 1. Bland Altman plot for intra observer reproducibilities of MM GLS


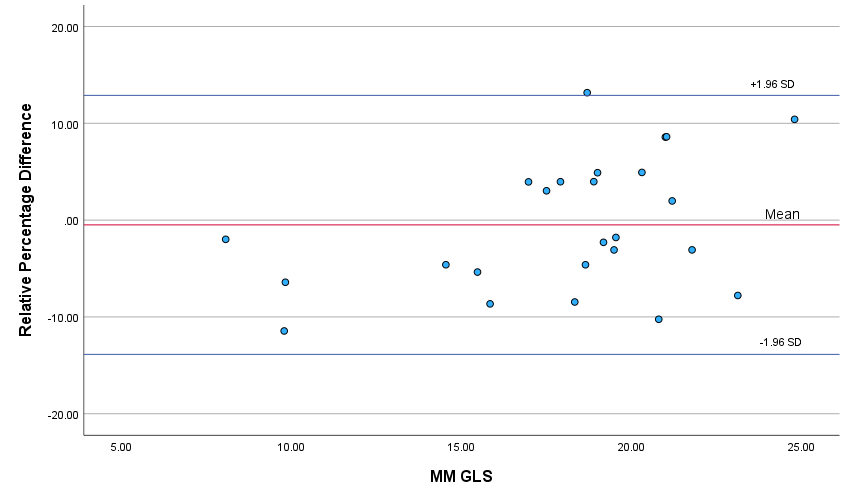


Supplementary Figure 2. Bland Altman plot for inter observer reproducibilities of MM GLS


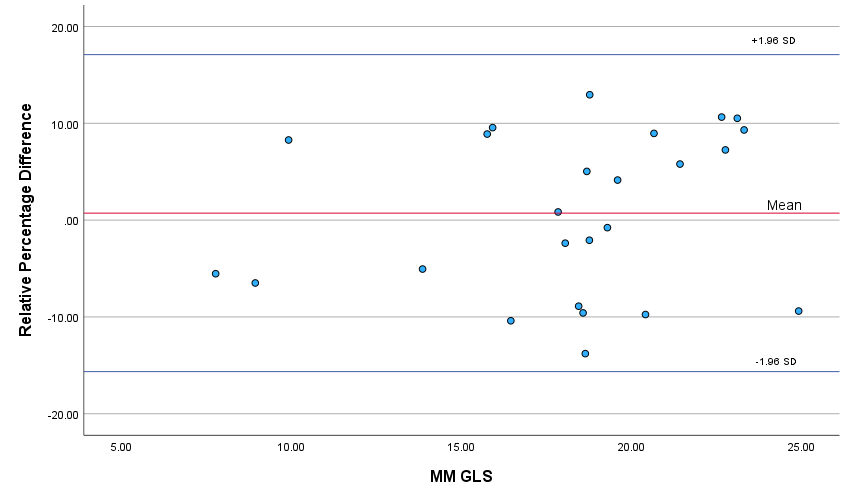

Supplement: Supplementary file 1 — Additional file 1: Fig. S1. Bland-Altman plot for intraobserver reproducibilities of MM-GLS. Fig. S2. Bland-Altman plot for interobserver reproducibilities of MM-GLS. [file 44348_2024_38_MOESM1_ESM.docx]
